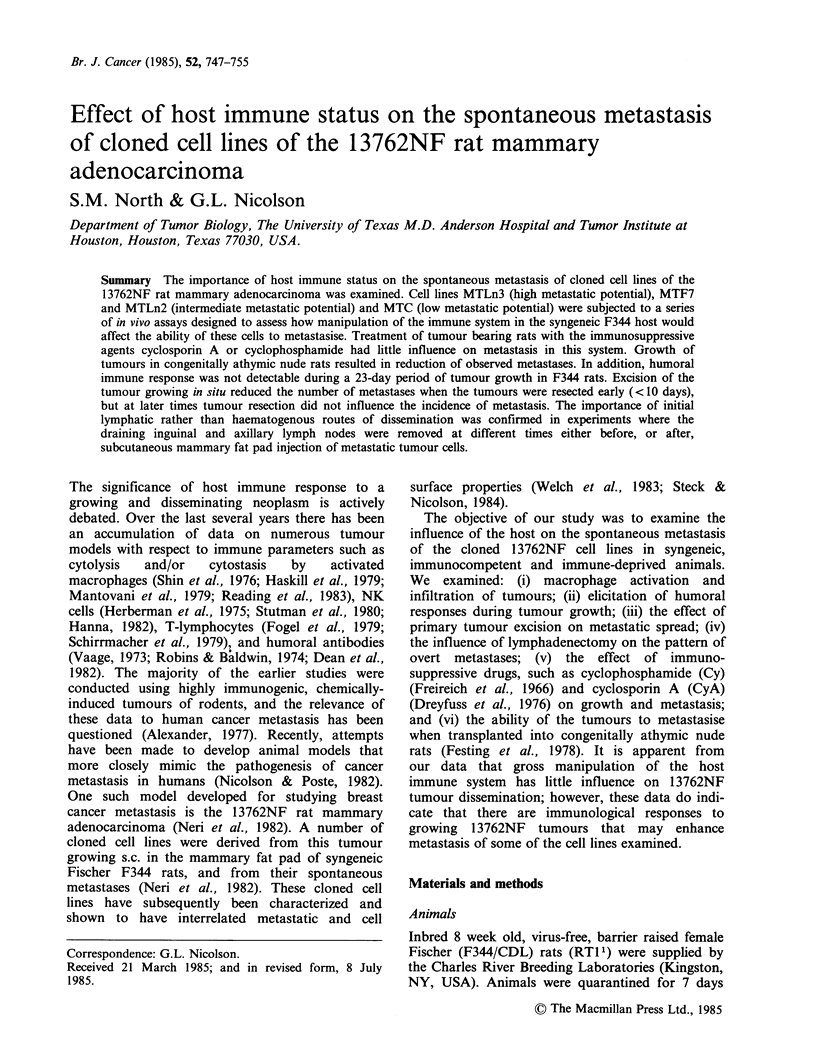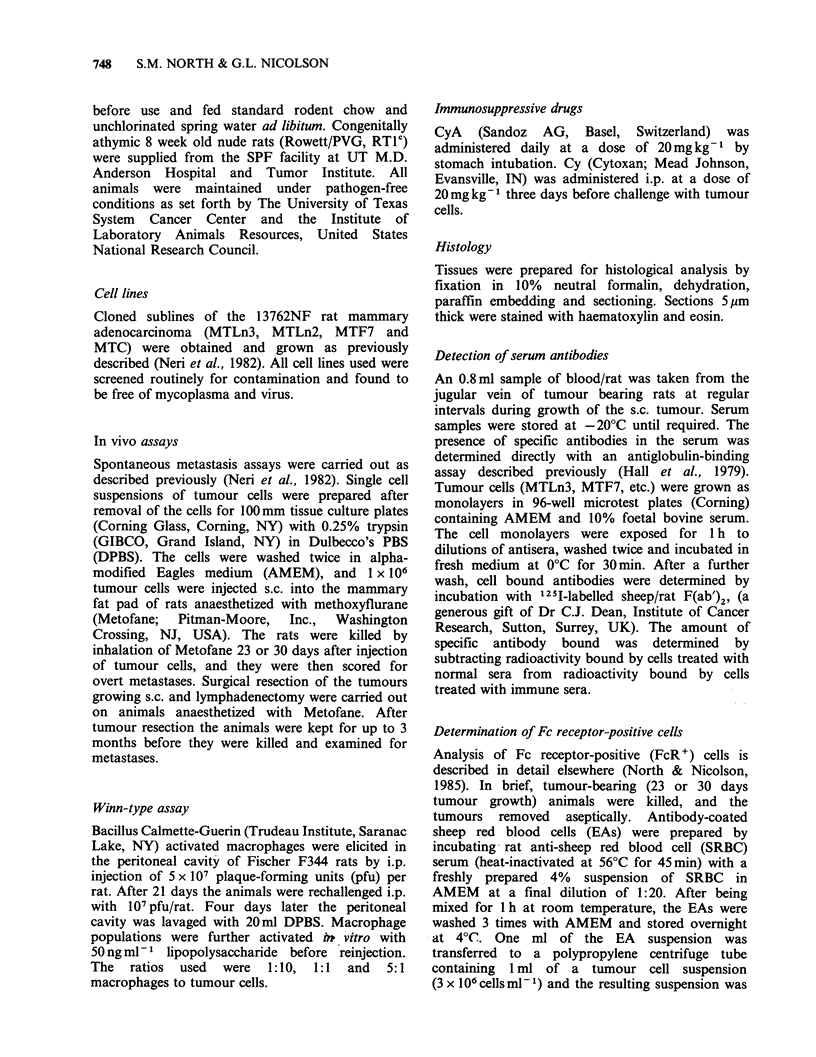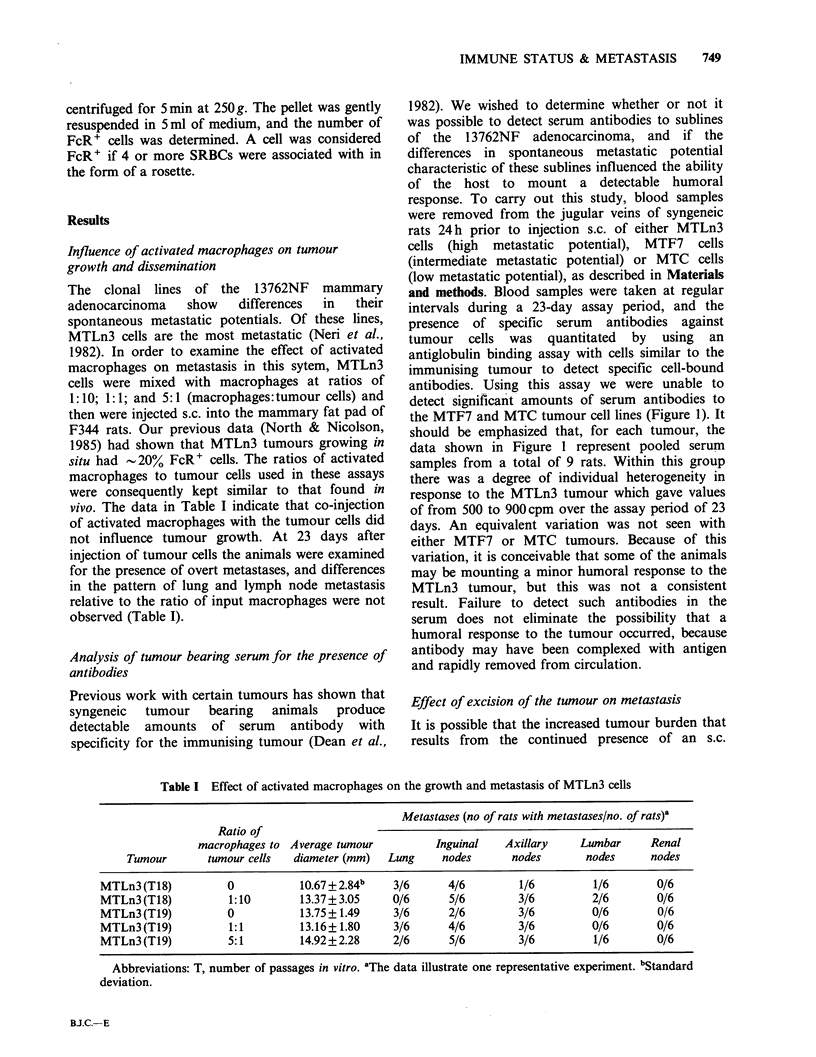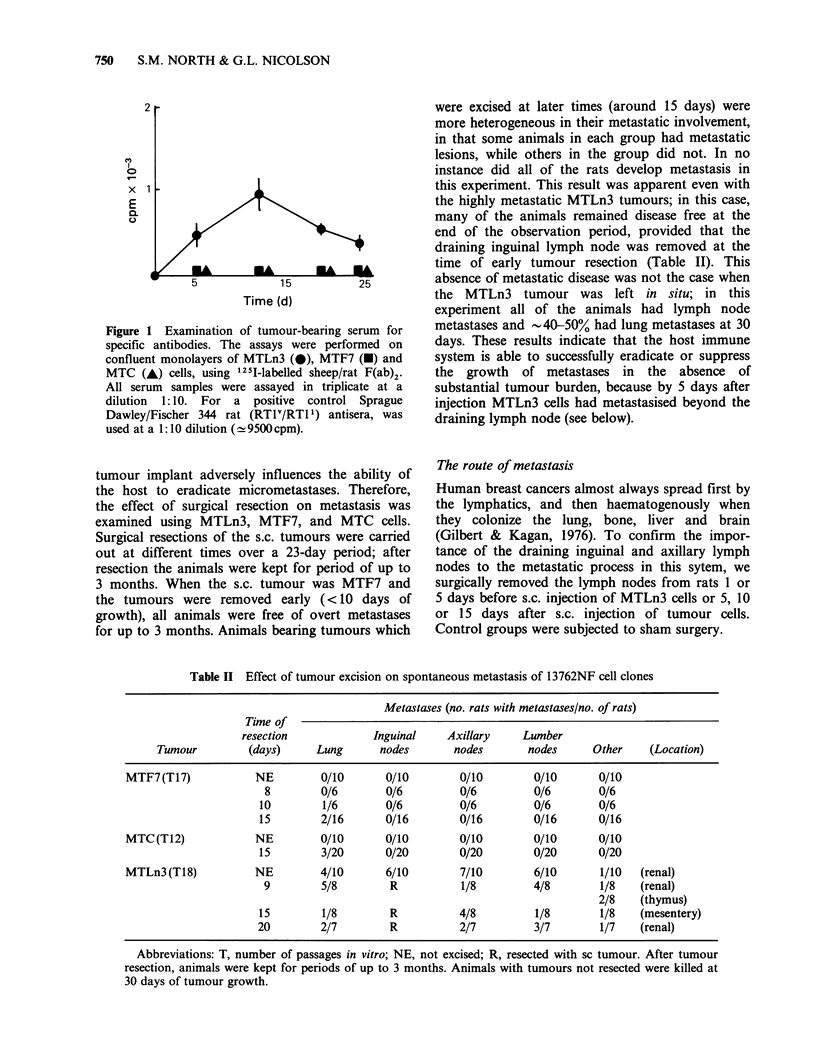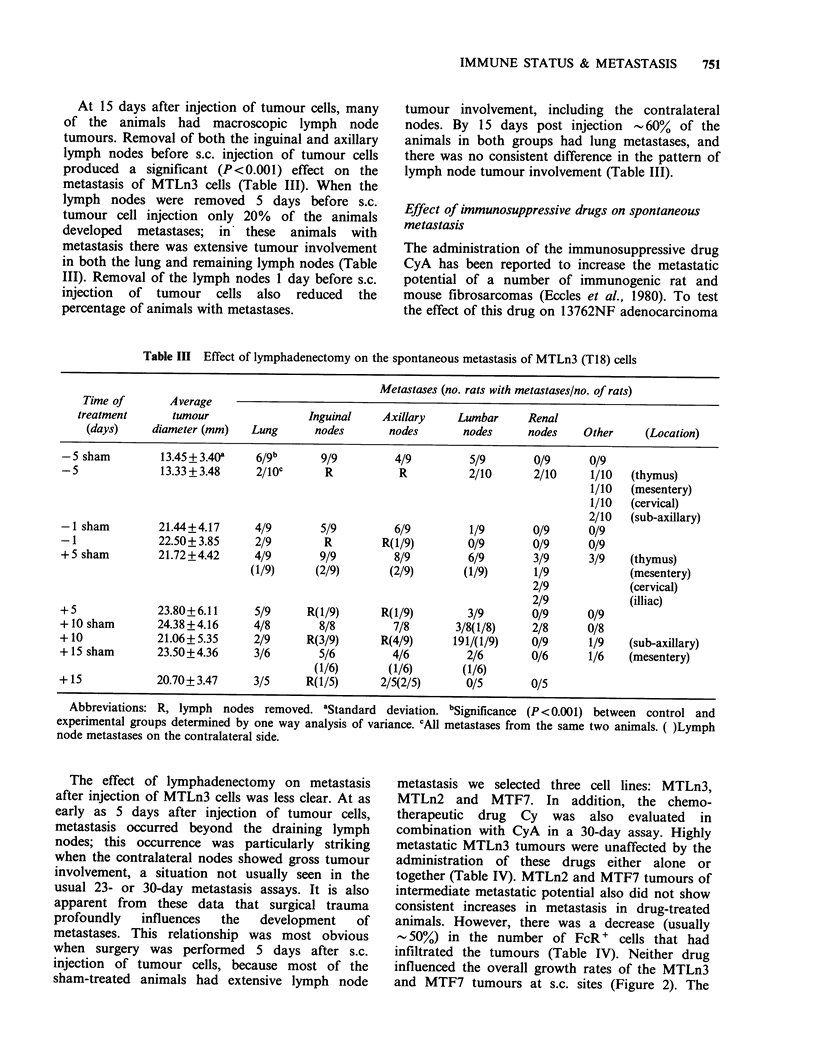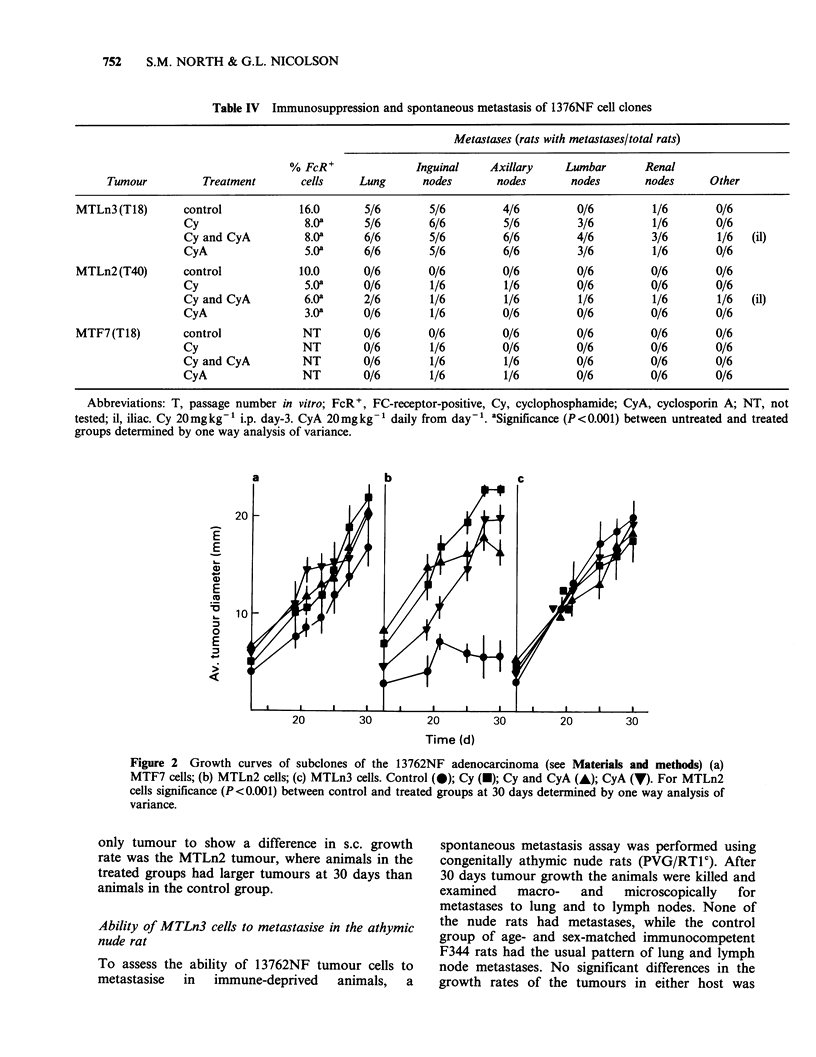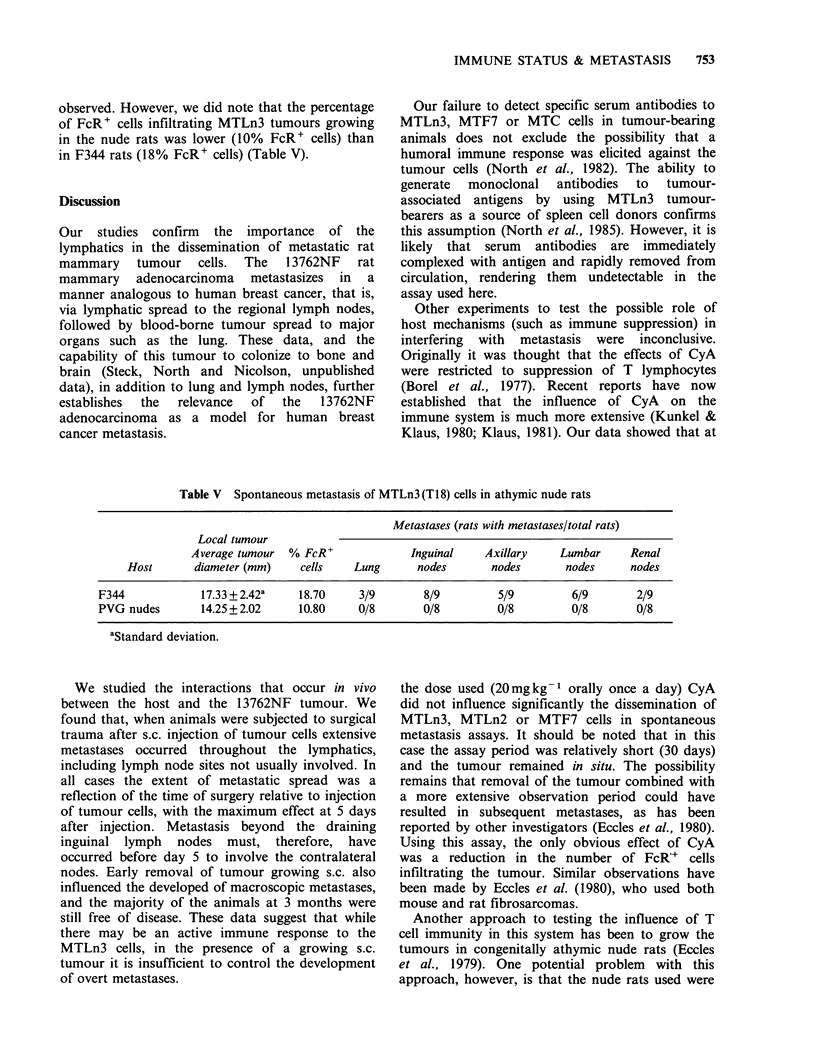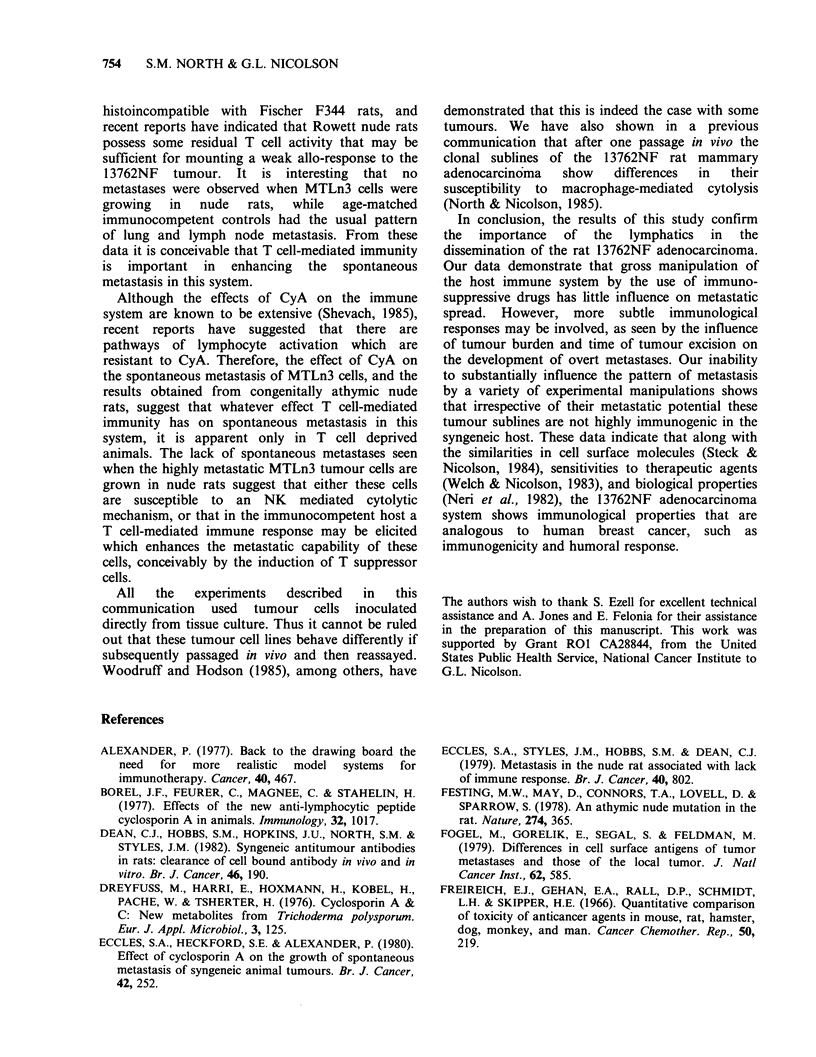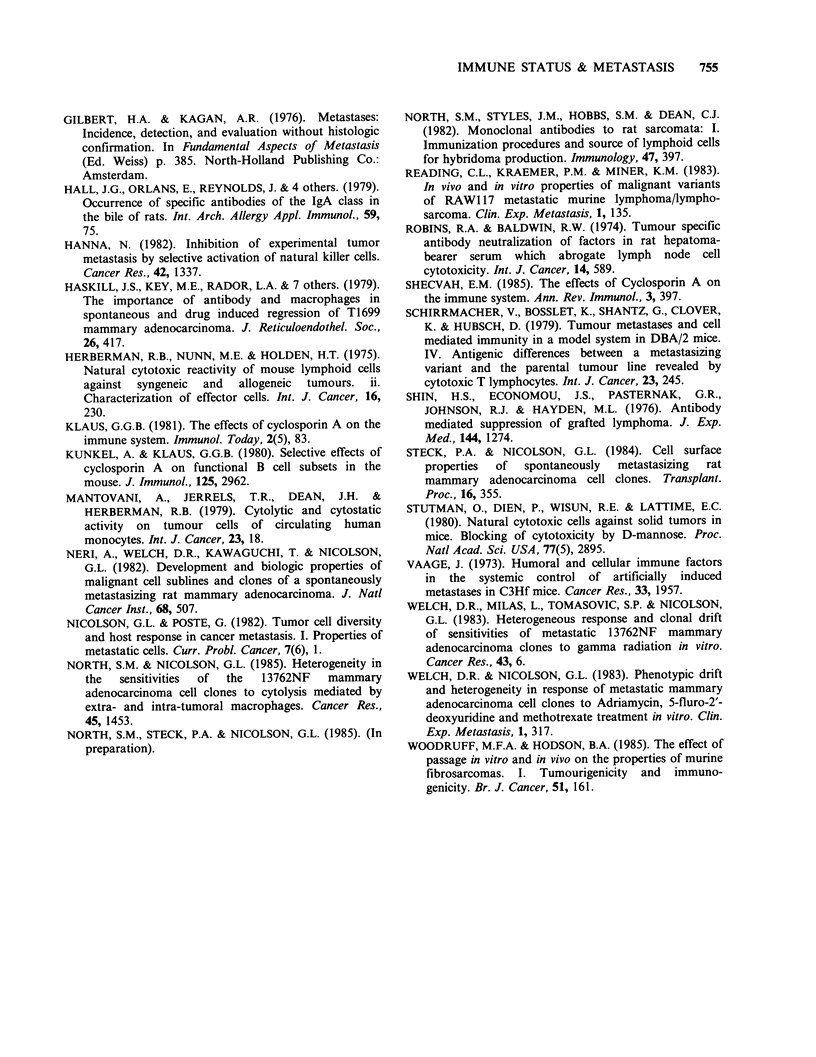# Effect of host immune status on the spontaneous metastasis of cloned cell lines of the 13762NF rat mammary adenocarcinoma.

**DOI:** 10.1038/bjc.1985.253

**Published:** 1985-11

**Authors:** S. M. North, G. L. Nicolson

## Abstract

The importance of host immune status on the spontaneous metastasis of cloned cell lines of the 13762NF rat mammary adenocarcinoma was examined. Cell lines MTLn3 (high metastatic potential), MTF7 and MTLn2 (intermediate metastatic potential) and MTC (low metastatic potential) were subjected to a series of in vivo assays designed to assess how manipulation of the immune system in the syngeneic F344 host would affect the ability of these cells to metastasise. Treatment of tumour bearing rats with the immunosuppressive agents cyclosporin A or cyclophosphamide had little influence on metastasis in this system. Growth of tumours in congenitally athymic nude rats resulted in reduction of observed metastases. In addition, humoral immune response was not detectable during a 23-day period of tumour growth in F344 rats. Excision of the tumour growing in situ reduced the number of metastases when the tumours were resected early (less than 10 days), but at later times tumour resection did not influence the incidence of metastasis. The importance of initial lymphatic rather than haematogenous routes of dissemination was confirmed in experiments where the draining inguinal and axillary lymph nodes were removed at different times either before, or after, subcutaneous mammary fat pad injection of metastatic tumour cells.


*Br. J. Cancer* (1985), **52**, 747–755

### Effect of host immune status on the spontaneous metastasis of cloned cell lines of the 13762NF rat mammary adenocarcinoma

S.M. North & G.L. Nicolson

*Department of Tumor Biology, The University of Texas M.D. Anderson Hospital and Tumor Institute at Houston, Houston, Texas 77030, USA.*

**Summary** The importance of host immune status on the spontaneous metastasis of cloned cell lines of the 13762NF rat mammary adenocarcinoma was examined. Cell lines MTLn3 (high metastatic potential), MTF7 and MTLn2 (intermediate metastatic potential) and MTC (low metastatic potential) were subjected to a series of *in vivo* assays designed to assess how manipulation of the immune system in the syngeneic F344 host would affect the ability of these cells to metastasise. Treatment of tumour bearing rats with the immunosuppressive agents cyclosporin A or cyclophosphamide had little influence on metastasis in this system. Growth of tumours in congenitally athymic nude rats resulted in reduction of observed metastases. In addition, humoral immune response was not detectable during a 23-day period of tumour growth in F344 rats. Excision of the tumour growing *in situ* reduced the number of metastases when the tumours were resected early (<10 days), but at later times tumour resection did not influence the incidence of metastasis. The importance of initial lymphatic rather than haematogenous routes of dissemination was confirmed in experiments where the draining inguinal and axillary lymph nodes were removed at different times either before, or after, subcutaneous mammary fat pad injection of metastatic tumour cells.

The significance of host immune response to a growing and disseminating neoplasm is actively debated. Over the last several years there has been an accumulation of data on numerous tumour models with respect to immune parameters such as cytolysis and/or cytostasis by activated macrophages (Shin *et al.*, 1976; Haskill *et al.*, 1979; Mantovani *et al.*, 1979; Reading *et al.*, 1983), NK cells (Herberman *et al.*, 1975; Stutman *et al.*, 1980; Hanna, 1982), T-lymphocytes (Fogel *et al.*, 1979; Schirrmacher *et al.*, 1979), and humoral antibodies (Vaage, 1973; Robins & Baldwin, 1974; Dean *et al.*, 1982). The majority of the earlier studies were conducted using highly immunogenic, chemically-induced tumours of rodents, and the relevance of these data to human cancer metastasis has been questioned (Alexander, 1977). Recently, attempts have been made to develop animal models that more closely mimic the pathogenesis of cancer metastasis in humans (Nicolson & Poste, 1982). One such model developed for studying breast cancer metastasis is the 13762NF rat mammary adenocarcinoma (Neri *et al.*, 1982). A number of cloned cell lines were derived from this tumour growing s.c. in the mammary fat pad of syngeneic Fischer F344 rats, and from their spontaneous metastases (Neri *et al.*, 1982). These cloned cell lines have subsequently been characterized and shown to have interrelated metastatic and cell surface properties (Welch *et al.*, 1983; Steck & Nicolson, 1984).

The objective of our study was to examine the influence of the host on the spontaneous metastasis of the cloned 13762NF cell lines in syngeneic, immunocompetent and immune-deprived animals. We examined: (i) macrophage activation and infiltration of tumours; (ii) elicitation of humoral responses during tumour growth; (iii) the effect of primary tumour excision on metastatic spread; (iv) the influence of lymphadenectomy on the pattern of overt metastases; (v) the effect of immunosuppressive drugs, such as cyclophosphamide (Cy) (Freireich *et al.*, 1966) and cyclosporin A (CyA) (Dreyfuss *et al.*, 1976) on growth and metastasis; and (vi) the ability of the tumours to metastasise when transplanted into congenitally athymic nude rats (Festing *et al.*, 1978). It is apparent from our data that gross manipulation of the host immune system has little influence on 13762NF tumour dissemination; however, these data do indicate that there are immunological responses to growing 13762NF tumours that may enhance metastasis of some of the cell lines examined.

### Materials and methods

### *Animals*

Inbred 8 week old, virus-free, barrier raised female Fischer (F344/CDL) rats (RT1[1]) were supplied by the Charles River Breeding Laboratories (Kingston, NY, USA). Animals were quarantined for 7 days

Correspondence: G.L. Nicolson.
Received 21 March 1985; and in revised form, 8 July 1985.

© The Macmillan Press Ltd., 1985

before use and fed standard rodent chow and unchlorinated spring water *ad libitum*. Congenitally athymic 8 week old nude rats (Rowett/PVG, $RT1^c$) were supplied from the SPF facility at UT M.D. Anderson Hospital and Tumor Institute. All animals were maintained under pathogen-free conditions as set forth by The University of Texas System Cancer Center and the Institute of Laboratory Animals Resources, United States National Research Council.

### *Cell lines*

Cloned sublines of the 13762NF rat mammary adenocarcinoma (MTLn3, MTLn2, MTF7 and MTC) were obtained and grown as previously described (Neri *et al.*, 1982). All cell lines used were screened routinely for contamination and found to be free of mycoplasma and virus.

### In vivo *assays*

Spontaneous metastasis assays were carried out as described previously (Neri *et al.*, 1982). Single cell suspensions of tumour cells were prepared after removal of the cells for 100 mm tissue culture plates (Corning Glass, Corning, NY) with 0.25% trypsin (GIBCO, Grand Island, NY) in Dulbecco's PBS (DPBS). The cells were washed twice in alpha-modified Eagles medium (AMEM), and $1 \times 10^6$ tumour cells were injected s.c. into the mammary fat pad of rats anaesthetized with methoxyflurane (Metofane; Pitman-Moore, Inc., Washington Crossing, NJ, USA). The rats were killed by inhalation of Metofane 23 or 30 days after injection of tumour cells, and they were then scored for overt metastases. Surgical resection of the tumours growing s.c. and lymphadenectomy were carried out on animals anaesthetized with Metofane. After tumour resection the animals were kept for up to 3 months before they were killed and examined for metastases.

### *Winn-type assay*

Bacillus Calmette-Guerin (Trudeau Institute, Saranac Lake, NY) activated macrophages were elicited in the peritoneal cavity of Fischer F344 rats by i.p. injection of $5 \times 10^7$ plaque-forming units (pfu) per rat. After 21 days the animals were rechallenged i.p. with $10^7$ pfu/rat. Four days later the peritoneal cavity was lavaged with 20 ml DPBS. Macrophage populations were further activated *in vitro* with 50 ng ml$^{-1}$ lipopolysaccharide before reinjection. The ratios used were 1:10, 1:1 and 5:1 macrophages to tumour cells.

### *Immunosuppressive drugs*

CyA (Sandoz AG, Basel, Switzerland) was administered daily at a dose of 20 mg kg$^{-1}$ by stomach intubation. Cy (Cytoxan; Mead Johnson, Evansville, IN) was administered i.p. at a dose of 20 mg kg$^{-1}$ three days before challenge with tumour cells.

### *Histology*

Tissues were prepared for histological analysis by fixation in 10% neutral formalin, dehydration, paraffin embedding and sectioning. Sections 5 $\mu$m thick were stained with haematoxylin and eosin.

### *Detection of serum antibodies*

An 0.8 ml sample of blood/rat was taken from the jugular vein of tumour bearing rats at regular intervals during growth of the s.c. tumour. Serum samples were stored at −20°C until required. The presence of specific antibodies in the serum was determined directly with an antiglobulin-binding assay described previously (Hall *et al.*, 1979). Tumour cells (MTLn3, MTF7, etc.) were grown as monolayers in 96-well microtest plates (Corning) containing AMEM and 10% foetal bovine serum. The cell monolayers were exposed for 1 h to dilutions of antisera, washed twice and incubated in fresh medium at 0°C for 30 min. After a further wash, cell bound antibodies were determined by incubation with $^{125}$I-labelled sheep/rat $F(ab')_2$, (a generous gift of Dr C.J. Dean, Institute of Cancer Research, Sutton, Surrey, UK). The amount of specific antibody bound was determined by subtracting radioactivity bound by cells treated with normal sera from radioactivity bound by cells treated with immune sera.

### *Determination of Fc receptor-positive cells*

Analysis of Fc receptor-positive ($FcR^+$) cells is described in detail elsewhere (North & Nicolson, 1985). In brief, tumour-bearing (23 or 30 days tumour growth) animals were killed, and the tumours removed aseptically. Antibody-coated sheep red blood cells (EAs) were prepared by incubating rat anti-sheep red blood cell (SRBC) serum (heat-inactivated at 56°C for 45 min) with a freshly prepared 4% suspension of SRBC in AMEM at a final dilution of 1:20. After being mixed for 1 h at room temperature, the EAs were washed 3 times with AMEM and stored overnight at 4°C. One ml of the EA suspension was transferred to a polypropylene centrifuge tube containing 1 ml of a tumour cell suspension ($3 \times 10^6$ cells ml$^{-1}$) and the resulting suspension was centrifuged for 5 min at 250 *g*. The pellet was gently resuspended in 5 ml of medium, and the number of $FcR^+$ cells was determined. A cell was considered $FcR^+$ if 4 or more SRBCs were associated with in the form of a rosette.

### Results

### *Influence of activated macrophages on tumour growth and dissemination*

The clonal lines of the 13762NF mammary adenocarcinoma show differences in their spontaneous metastatic potentials. Of these lines, MTLn3 cells are the most metastatic (Neri *et al.*, 1982). In order to examine the effect of activated macrophages on metastasis in this sytem, MTLn3 cells were mixed with macrophages at ratios of 1:10; 1:1; and 5:1 (macrophages:tumour cells) and then were injected s.c. into the mammary fat pad of F344 rats. Our previous data (North & Nicolson, 1985) had shown that MTLn3 tumours growing *in situ* had ~20% $FcR^+$ cells. The ratios of activated macrophages to tumour cells used in these assays were consequently kept similar to that found *in vivo*. The data in Table I indicate that co-injection of activated macrophages with the tumour cells did not influence tumour growth. At 23 days after injection of tumour cells the animals were examined for the presence of overt metastases, and differences in the pattern of lung and lymph node metastasis relative to the ratio of input macrophages were not observed (Table I).

### *Analysis of tumour bearing serum for the presence of antibodies*

Previous work with certain tumours has shown that syngeneic tumour bearing animals produce detectable amounts of serum antibody with specificity for the immunising tumour (Dean *et al.*, 1982). We wished to determine whether or not it was possible to detect serum antibodies to sublines of the 13762NF adenocarcinoma, and if the differences in spontaneous metastatic potential characteristic of these sublines influenced the ability of the host to mount a detectable humoral response. To carry out this study, blood samples were removed from the jugular veins of syngeneic rats 24 h prior to injection s.c. of either MTLn3 cells (high metastatic potential), MTF7 cells (intermediate metastatic potential) or MTC cells (low metastatic potential), as described in **Materials and methods**. Blood samples were taken at regular intervals during a 23-day assay period, and the presence of specific serum antibodies against tumour cells was quantitated by using an antiglobulin binding assay with cells similar to the immunising tumour to detect specific cell-bound antibodies. Using this assay we were unable to detect significant amounts of serum antibodies to the MTF7 and MTC tumour cell lines (Figure 1). It should be emphasized that, for each tumour, the data shown in Figure 1 represent pooled serum samples from a total of 9 rats. Within this group there was a degree of individual heterogeneity in response to the MTLn3 tumour which gave values of from 500 to 900 cpm over the assay period of 23 days. An equivalent variation was not seen with either MTF7 or MTC tumours. Because of this variation, it is conceivable that some of the animals may be mounting a minor humoral response to the MTLn3 tumour, but this was not a consistent result. Failure to detect such antibodies in the serum does not eliminate the possibility that a humoral response to the tumour occurred, because antibody may have been complexed with antigen and rapidly removed from circulation.

### *Effect of excision of the tumour on metastasis*

It is possible that the increased tumour burden that results from the continued presence of an s.c.

**Table I** Effect of activated macrophages on the growth and metastasis of MTLn3 cells

| Tumour | Ratio of macrophages to tumour cells | Average tumour diameter (mm) | Metastases (no of rats with metastases/no. of rats)[a] Lung | Inguinal nodes | Axillary nodes | Lumbar nodes | Renal nodes |
|---|---|---|---|---|---|---|---|
| MTLn3 (T18) | 0 | 10.67 ± 2.84[b] | 3/6 | 4/6 | 1/6 | 1/6 | 0/6 |
| MTLn3 (T18) | 1:10 | 13.37 ± 3.05 | 0/6 | 5/6 | 3/6 | 2/6 | 0/6 |
| MTLn3 (T19) | 0 | 13.75 ± 1.49 | 3/6 | 2/6 | 3/6 | 0/6 | 0/6 |
| MTLn3 (T19) | 1:1 | 13.16 ± 1.80 | 3/6 | 4/6 | 3/6 | 0/6 | 0/6 |
| MTLn3 (T19) | 5:1 | 14.92 ± 2.28 | 2/6 | 5/6 | 3/6 | 1/6 | 0/6 |

Abbreviations: T, number of passages *in vitro*. [a]The data illustrate one representative experiment. [b]Standard deviation.

**Figure 1** Examination of tumour-bearing serum for specific antibodies. The assays were performed on confluent monolayers of MTLn3 (●), MTF7 (■) and MTC (▲) cells, using $^{125}$I-labelled sheep/rat $F(ab)_2$. All serum samples were assayed in triplicate at a dilution 1:10. For a positive control Sprague Dawley/Fischer 344 rat ($RT1^v/RT1^1$) antisera, was used at a 1:10 dilution (≃9500 cpm).

tumour implant adversely influences the ability of the host to eradicate micrometastases. Therefore, the effect of surgical resection on metastasis was examined using MTLn3, MTF7, and MTC cells. Surgical resections of the s.c. tumours were carried out at different times over a 23-day period; after resection the animals were kept for period of up to 3 months. When the s.c. tumour was MTF7 and the tumours were removed early (<10 days of growth), all animals were free of overt metastases for up to 3 months. Animals bearing tumours which were excised at later times (around 15 days) were more heterogeneous in their metastatic involvement, in that some animals in each group had metastatic lesions, while others in the group did not. In no instance did all of the rats develop metastasis in this experiment. This result was apparent even with the highly metastatic MTLn3 tumours; in this case, many of the animals remained disease free at the end of the observation period, provided that the draining inguinal lymph node was removed at the time of early tumour resection (Table II). This absence of metastatic disease was not the case when the MTLn3 tumour was left *in situ*; in this experiment all of the animals had lymph node metastases and ~40–50% had lung metastases at 30 days. These results indicate that the host immune system is able to successfully eradicate or suppress the growth of metastases in the absence of substantial tumour burden, because by 5 days after injection MTLn3 cells had metastasised beyond the draining lymph node (see below).

### *The route of metastasis*

Human breast cancers almost always spread first by the lymphatics, and then haematogenously when they colonize the lung, bone, liver and brain (Gilbert & Kagan, 1976). To confirm the importance of the draining inguinal and axillary lymph nodes to the metastatic process in this sytem, we surgically removed the lymph nodes from rats 1 or 5 days before s.c. injection of MTLn3 cells or 5, 10 or 15 days after s.c. injection of tumour cells. Control groups were subjected to sham surgery.

**Table II** Effect of tumour excision on spontaneous metastasis of 13762NF cell clones

| | | *Metastases (no. rats with metastases/no. of rats)* | | | | | |
|---|---|---|---|---|---|---|---|
| *Tumour* | *Time of resection (days)* | *Lung* | *Inguinal nodes* | *Axillary nodes* | *Lumber nodes* | *Other* | *(Location)* |
| MTF7(T17) | NE | 0/10 | 0/10 | 0/10 | 0/10 | 0/10 | |
| | 8 | 0/6 | 0/6 | 0/6 | 0/6 | 0/6 | |
| | 10 | 1/6 | 0/6 | 0/6 | 0/6 | 0/6 | |
| | 15 | 2/16 | 0/16 | 0/16 | 0/16 | 0/16 | |
| MTC(T12) | NE | 0/10 | 0/10 | 0/10 | 0/10 | 0/10 | |
| | 15 | 3/20 | 0/20 | 0/20 | 0/20 | 0/20 | |
| MTLn3(T18) | NE | 4/10 | 6/10 | 7/10 | 6/10 | 1/10 | (renal) |
| | 9 | 5/8 | R | 1/8 | 4/8 | 1/8 | (renal) |
| | | | | | | 2/8 | (thymus) |
| | 15 | 1/8 | R | 4/8 | 1/8 | 1/8 | (mesentery) |
| | 20 | 2/7 | R | 2/7 | 3/7 | 1/7 | (renal) |

Abbreviations: T, number of passages *in vitro*; NE, not excised; R, resected with sc tumour. After tumour resection, animals were kept for periods of up to 3 months. Animals with tumours not resected were killed at 30 days of tumour growth.

At 15 days after injection of tumour cells, many of the animals had macroscopic lymph node tumours. Removal of both the inguinal and axillary lymph nodes before s.c. injection of tumour cells produced a significant ($P<0.001$) effect on the metastasis of MTLn3 cells (Table III). When the lymph nodes were removed 5 days before s.c. tumour cell injection only 20% of the animals developed metastases; in these animals with metastasis there was extensive tumour involvement in both the lung and remaining lymph nodes (Table III). Removal of the lymph nodes 1 day before s.c. injection of tumour cells also reduced the percentage of animals with metastases.

The effect of lymphadenectomy on metastasis after injection of MTLn3 cells was less clear. At as early as 5 days after injection of tumour cells, metastasis occurred beyond the draining lymph nodes; this occurrence was particularly striking when the contralateral nodes showed gross tumour involvement, a situation not usually seen in the usual 23- or 30-day metastasis assays. It is also apparent from these data that surgical trauma profoundly influences the development of metastases. This relationship was most obvious when surgery was performed 5 days after s.c. injection of tumour cells, because most of the sham-treated animals had extensive lymph node tumour involvement, including the contralateral nodes. By 15 days post injection ~60% of the animals in both groups had lung metastases, and there was no consistent difference in the pattern of lymph node tumour involvement (Table III).

**Table III** Effect of lymphadenectomy on the spontaneous metastasis of MTLn3 (T18) cells

| *Time of treatment (days)* | *Average tumour diameter (mm)* | *Metastases (no. rats with metastases/no. of rats)* | | | | | | |
|---|---|---|---|---|---|---|---|---|
| | | *Lung* | *Inguinal nodes* | *Axillary nodes* | *Lumbar nodes* | *Renal nodes* | *Other* | *(Location)* |
| −5 sham | 13.45±3.40[a] | 6/9[b] | 9/9 | 4/9 | 5/9 | 0/9 | 0/9 | |
| −5 | 13.33±3.48 | 2/10[c] | R | R | 2/10 | 2/10 | 1/10 | (thymus) |
| | | | | | | | 1/10 | (mesentery) |
| | | | | | | | 1/10 | (cervical) |
| | | | | | | | 2/10 | (sub-axillary) |
| −1 sham | 21.44±4.17 | 4/9 | 5/9 | 6/9 | 1/9 | 0/9 | 0/9 | |
| −1 | 22.50±3.85 | 2/9 | R | R(1/9) | 0/9 | 0/9 | 0/9 | |
| +5 sham | 21.72±4.42 | 4/9 | 9/9 | 8/9 | 6/9 | 3/9 | 3/9 | (thymus) |
| | | (1/9) | (2/9) | (2/9) | (1/9) | 1/9 | | (mesentery) |
| | | | | | | 2/9 | | (cervical) |
| | | | | | | 2/9 | | (illiac) |
| +5 | 23.80±6.11 | 5/9 | R(1/9) | R(1/9) | 3/9 | 0/9 | 0/9 | |
| +10 sham | 24.38±4.16 | 4/8 | 8/8 | 7/8 | 3/8(1/8) | 2/8 | 0/8 | |
| +10 | 21.06±5.35 | 2/9 | R(3/9) | R(4/9) | 191/(1/9) | 0/9 | 1/9 | (sub-axillary) |
| +15 sham | 23.50±4.36 | 3/6 | 5/6 | 4/6 | 2/6 | 0/6 | 1/6 | (mesentery) |
| | | | (1/6) | (1/6) | (1/6) | | | |
| +15 | 20.70±3.47 | 3/5 | R(1/5) | 2/5(2/5) | 0/5 | 0/5 | | |

Abbreviations: R, lymph nodes removed. [a]Standard deviation. [b]Significance ($P<0.001$) between control and experimental groups determined by one way analysis of variance. [c]All metastases from the same two animals. ( )Lymph node metastases on the contralateral side.

*Effect of immunosuppressive drugs on spontaneous metastasis*

The administration of the immunosuppressive drug CyA has been reported to increase the metastatic potential of a number of immunogenic rat and mouse fibrosarcomas (Eccles *et al.*, 1980). To test the effect of this drug on 13762NF adenocarcinoma metastasis we selected three cell lines: MTLn3, MTLn2 and MTF7. In addition, the chemotherapeutic drug Cy was also evaluated in combination with CyA in a 30-day assay. Highly metastatic MTLn3 tumours were unaffected by the administration of these drugs either alone or together (Table IV). MTLn2 and MTF7 tumours of intermediate metastatic potential also did not show consistent increases in metastasis in drug-treated animals. However, there was a decrease (usually ~50%) in the number of $FcR^+$ cells that had infiltrated the tumours (Table IV). Neither drug influenced the overall growth rates of the MTLn3 and MTF7 tumours at s.c. sites (Figure 2). The

**Table IV** Immunosuppression and spontaneous metastasis of 1376NF cell clones

| | | | *Metastases (rats with metastases/total rats)* | | | | | |
|---|---|---|---|---|---|---|---|---|
| *Tumour* | *Treatment* | *% FcR⁺ cells* | *Lung* | *Inguinal nodes* | *Axillary nodes* | *Lumbar nodes* | *Renal nodes* | *Other* |
| MTLn3(T18) | control | 16.0 | 5/6 | 5/6 | 4/6 | 0/6 | 1/6 | 0/6 |
| | Cy | 8.0[a] | 5/6 | 6/6 | 5/6 | 3/6 | 1/6 | 0/6 |
| | Cy and CyA | 8.0[a] | 6/6 | 5/6 | 6/6 | 4/6 | 3/6 | 1/6 (il) |
| | CyA | 5.0[a] | 6/6 | 5/6 | 6/6 | 3/6 | 1/6 | 0/6 |
| MTLn2(T40) | control | 10.0 | 0/6 | 0/6 | 0/6 | 0/6 | 0/6 | 0/6 |
| | Cy | 5.0[a] | 0/6 | 1/6 | 1/6 | 0/6 | 0/6 | 0/6 |
| | Cy and CyA | 6.0[a] | 2/6 | 1/6 | 1/6 | 1/6 | 1/6 | 1/6 (il) |
| | CyA | 3.0[a] | 0/6 | 1/6 | 0/6 | 0/6 | 0/6 | 0/6 |
| MTF7(T18) | control | NT | 0/6 | 0/6 | 0/6 | 0/6 | 0/6 | 0/6 |
| | Cy | NT | 0/6 | 1/6 | 0/6 | 0/6 | 0/6 | 0/6 |
| | Cy and CyA | NT | 0/6 | 1/6 | 1/6 | 0/6 | 0/6 | 0/6 |
| | CyA | NT | 0/6 | 1/6 | 1/6 | 0/6 | 0/6 | 0/6 |

Abbreviations: T, passage number *in vitro*; FcR⁺, FC-receptor-positive, Cy, cyclophosphamide; CyA, cyclosporin A; NT, not tested; il, iliac. Cy 20 mg kg$^{-1}$ i.p. day-3. CyA 20 mg kg$^{-1}$ daily from day$^{-1}$. [a]Significance ($P < 0.001$) between untreated and treated groups determined by one way analysis of variance.

**Figure 2** Growth curves of subclones of the 13762NF adenocarcinoma (see **Materials and methods**) (a) MTF7 cells; (b) MTLn2 cells; (c) MTLn3 cells. Control (●); Cy (■); Cy and CyA (▲); CyA (▼). For MTLn2 cells significance ($P < 0.001$) between control and treated groups at 30 days determined by one way analysis of variance.

only tumour to show a difference in s.c. growth rate was the MTLn2 tumour, where animals in the treated groups had larger tumours at 30 days than animals in the control group.

*Ability of MTLn3 cells to metastasise in the athymic nude rat*

To assess the ability of 13762NF tumour cells to metastasise in immune-deprived animals, a spontaneous metastasis assay was performed using congenitally athymic nude rats (PVG/RT1[c]). After 30 days tumour growth the animals were killed and examined macro- and microscopically for metastases to lung and to lymph nodes. None of the nude rats had metastases, while the control group of age- and sex-matched immunocompetent F344 rats had the usual pattern of lung and lymph node metastases. No significant differences in the growth rates of the tumours in either host was observed. However, we did note that the percentage of FcR$^+$ cells infiltrating MTLn3 tumours growing in the nude rats was lower (10% FcR$^+$ cells) than in F344 rats (18% FcR$^+$ cells) (Table V).

### Discussion

Our studies confirm the importance of the lymphatics in the dissemination of metastatic rat mammary tumour cells. The 13762NF rat mammary adenocarcinoma metastasizes in a manner analogous to human breast cancer, that is, via lymphatic spread to the regional lymph nodes, followed by blood-borne tumour spread to major organs such as the lung. These data, and the capability of this tumour to colonize to bone and brain (Steck, North and Nicolson, unpublished data), in addition to lung and lymph nodes, further establishes the relevance of the 13762NF adenocarcinoma as a model for human breast cancer metastasis.

Our failure to detect specific serum antibodies to MTLn3, MTF7 or MTC cells in tumour-bearing animals does not exclude the possibility that a humoral immune response was elicited against the tumour cells (North *et al.*, 1982). The ability to generate monoclonal antibodies to tumour-associated antigens by using MTLn3 tumour-bearers as a source of spleen cell donors confirms this assumption (North *et al.*, 1985). However, it is likely that serum antibodies are immediately complexed with antigen and rapidly removed from circulation, rendering them undetectable in the assay used here.

Other experiments to test the possible role of host mechanisms (such as immune suppression) in interfering with metastasis were inconclusive. Originally it was thought that the effects of CyA were restricted to suppression of T lymphocytes (Borel *et al.*, 1977). Recent reports have now established that the influence of CyA on the immune system is much more extensive (Kunkel & Klaus, 1980; Klaus, 1981). Our data showed that at

**Table V** Spontaneous metastasis of MTLn3(T18) cells in athymic nude rats

| *Host* | *Local tumour Average tumour diameter (mm)* | *% FcR$^+$ cells* | *Metastases (rats with metastases/total rats)* | | | | |
|---|---|---|---|---|---|---|---|
| | | | *Lung* | *Inguinal nodes* | *Axillary nodes* | *Lumbar nodes* | *Renal nodes* |
| F344 | 17.33±2.42[a] | 18.70 | 3/9 | 8/9 | 5/9 | 6/9 | 2/9 |
| PVG nudes | 14.25±2.02 | 10.80 | 0/8 | 0/8 | 0/8 | 0/8 | 0/8 |

[a]Standard deviation.

We studied the interactions that occur *in vivo* between the host and the 13762NF tumour. We found that, when animals were subjected to surgical trauma after s.c. injection of tumour cells extensive metastases occurred throughout the lymphatics, including lymph node sites not usually involved. In all cases the extent of metastatic spread was a reflection of the time of surgery relative to injection of tumour cells, with the maximum effect at 5 days after injection. Metastasis beyond the draining inguinal lymph nodes must, therefore, have occurred before day 5 to involve the contralateral nodes. Early removal of tumour growing s.c. also influenced the developed of macroscopic metastases, and the majority of the animals at 3 months were still free of disease. These data suggest that while there may be an active immune response to the MTLn3 cells, in the presence of a growing s.c. tumour it is insufficient to control the development of overt metastases.

the dose used (20 mg kg$^{-1}$ orally once a day) CyA did not influence significantly the dissemination of MTLn3, MTLn2 or MTF7 cells in spontaneous metastasis assays. It should be noted that in this case the assay period was relatively short (30 days) and the tumour remained *in situ*. The possibility remains that removal of the tumour combined with a more extensive observation period could have resulted in subsequent metastases, as has been reported by other investigators (Eccles *et al.*, 1980). Using this assay, the only obvious effect of CyA was a reduction in the number of FcR$^+$ cells infiltrating the tumour. Similar observations have been made by Eccles *et al.* (1980), who used both mouse and rat fibrosarcomas.

Another approach to testing the influence of T cell immunity in this system has been to grow the tumours in congenitally athymic nude rats (Eccles *et al.*, 1979). One potential problem with this approach, however, is that the nude rats used were histoincompatible with Fischer F344 rats, and recent reports have indicated that Rowett nude rats possess some residual T cell activity that may be sufficient for mounting a weak allo-response to the 13762NF tumour. It is interesting that no metastases were observed when MTLn3 cells were growing in nude rats, while age-matched immunocompetent controls had the usual pattern of lung and lymph node metastasis. From these data it is conceivable that T cell-mediated immunity is important in enhancing the spontaneous metastasis in this system.

Although the effects of CyA on the immune system are known to be extensive (Shevach, 1985), recent reports have suggested that there are pathways of lymphocyte activation which are resistant to CyA. Therefore, the effect of CyA on the spontaneous metastasis of MTLn3 cells, and the results obtained from congenitally athymic nude rats, suggest that whatever effect T cell-mediated immunity has on spontaneous metastasis in this system, it is apparent only in T cell deprived animals. The lack of spontaneous metastases seen when the highly metastatic MTLn3 tumour cells are grown in nude rats suggest that either these cells are susceptible to an NK mediated cytolytic mechanism, or that in the immunocompetent host a T cell-mediated immune response may be elicited which enhances the metastatic capability of these cells, conceivably by the induction of T suppressor cells.

All the experiments described in this communication used tumour cells inoculated directly from tissue culture. Thus it cannot be ruled out that these tumour cell lines behave differently if subsequently passaged *in vivo* and then reassayed. Woodruff and Hodson (1985), among others, have demonstrated that this is indeed the case with some tumours. We have also shown in a previous communication that after one passage *in vivo* the clonal sublines of the 13762NF rat mammary adenocarcinoma show differences in their susceptibility to macrophage-mediated cytolysis (North & Nicolson, 1985).

In conclusion, the results of this study confirm the importance of the lymphatics in the dissemination of the rat 13762NF adenocarcinoma. Our data demonstrate that gross manipulation of the host immune system by the use of immunosuppressive drugs has little influence on metastatic spread. However, more subtle immunological responses may be involved, as seen by the influence of tumour burden and time of tumour excision on the development of overt metastases. Our inability to substantially influence the pattern of metastasis by a variety of experimental manipulations shows that irrespective of their metastatic potential these tumour sublines are not highly immunogenic in the syngeneic host. These data indicate that along with the similarities in cell surface molecules (Steck & Nicolson, 1984), sensitivities to therapeutic agents (Welch & Nicolson, 1983), and biological properties (Neri *et al.*, 1982), the 13762NF adenocarcinoma system shows immunological properties that are analogous to human breast cancer, such as immunogenicity and humoral response.

The authors wish to thank S. Ezell for excellent technical assistance and A. Jones and E. Felonia for their assistance in the preparation of this manuscript. This work was supported by Grant RO1 CA28844, from the United States Public Health Service, National Cancer Institute to G.L. Nicolson.

### References

ALEXANDER, P. (1977). Back to the drawing board the need for more realistic model systems for immunotherapy. *Cancer*, **40**, 467.

BOREL, J.F., FEURER, C., MAGNEE, C. & STAHELIN, H. (1977). Effects of the new anti-lymphocytic peptide cyclosporin A in animals. *Immunology*, **32**, 1017.

DEAN, C.J., HOBBS, S.M., HOPKINS, J.U., NORTH, S.M. & STYLES, J.M. (1982). Syngeneic antitumour antibodies in rats: clearance of cell bound antibody *in vivo* and *in vitro*. *Br. J. Cancer*, **46**, 190.

DREYFUSS, M., HARRI, E., HOXMANN, H., KOBEL, H., PACHE, W. & TSHERTER, H. (1976). Cyclosporin A & C: New metabolites from *Trichoderma polysporum*. *Eur. J. Appl. Microbiol.*, **3**, 125.

ECCLES, S.A., HECKFORD, S.E. & ALEXANDER, P. (1980). Effect of cyclosporin A on the growth of spontaneous metastasis of syngeneic animal tumours. *Br. J. Cancer*, **42**, 252.

ECCLES, S.A., STYLES, J.M., HOBBS, S.M. & DEAN, C.J. (1979). Metastasis in the nude rat associated with lack of immune response. *Br. J. Cancer*, **40**, 802.

FESTING, M.W., MAY, D., CONNORS, T.A., LOVELL, D. & SPARROW, S. (1978). An athymic nude mutation in the rat. *Nature*, **274**, 365.

FOGEL, M., GORELIK, E., SEGAL, S. & FELDMAN, M. (1979). Differences in cell surface antigens of tumor metastases and those of the local tumor. *J. Natl Cancer Inst.*, **62**, 585.

FREIREICH, E.J., GEHAN, E.A., RALL, D.P., SCHMIDT, L.H. & SKIPPER, H.E. (1966). Quantitative comparison of toxicity of anticancer agents in mouse, rat, hamster, dog, monkey, and man. *Cancer Chemother. Rep.*, **50**, 219.

GILBERT, H.A. & KAGAN, A.R. (1976). Metastases: Incidence, detection, and evaluation without histologic confirmation. In *Fundamental Aspects of Metastasis* (Ed. Weiss) p. 385. North-Holland Publishing Co.: Amsterdam.

HALL, J.G., ORLANS, E., REYNOLDS, J. & 4 others. (1979). Occurrence of specific antibodies of the IgA class in the bile of rats. *Int. Arch. Allergy Appl. Immunol.*, **59**, 75.

HANNA, N. (1982). Inhibition of experimental tumor metastasis by selective activation of natural killer cells. *Cancer Res.*, **42**, 1337.

HASKILL, J.S., KEY, M.E., RADOR, L.A. & 7 others. (1979). The importance of antibody and macrophages in spontaneous and drug induced regression of T1699 mammary adenocarcinoma. *J. Reticuloendothel. Soc.*, **26**, 417.

HERBERMAN, R.B., NUNN, M.E. & HOLDEN, H.T. (1975). Natural cytotoxic reactivity of mouse lymphoid cells against syngeneic and allogeneic tumours. ii. Characterization of effector cells. *Int. J. Cancer*, **16**, 230.

KLAUS, G.G.B. (1981). The effects of cyclosporin A on the immune system. *Immunol. Today*, **2**(5), 83.

KUNKEL, A. & KLAUS, G.G.B. (1980). Selective effects of cyclosporin A on functional B cell subsets in the mouse. *J. Immunol.*, **125**, 2962.

MANTOVANI, A., JERRELS, T.R., DEAN, J.H. & HERBERMAN, R.B. (1979). Cytolytic and cytostatic activity on tumour cells of circulating human monocytes. *Int. J. Cancer*, **23**, 18.

NERI, A., WELCH, D.R., KAWAGUCHI, T. & NICOLSON, G.L. (1982). Development and biologic properties of malignant cell sublines and clones of a spontaneously metastasizing rat mammary adenocarcinoma. *J. Natl Cancer Inst.*, **68**, 507.

NICOLSON, G.L. & POSTE, G. (1982). Tumor cell diversity and host response in cancer metastasis. I. Properties of metastatic cells. *Curr. Probl. Cancer*, **7**(6), 1.

NORTH, S.M. & NICOLSON, G.L. (1985). Heterogeneity in the sensitivities of the 13762NF mammary adenocarcinoma cell clones to cytolysis mediated by extra- and intra-tumoral macrophages. *Cancer Res.*, **45**, 1453.

NORTH, S.M., STECK, P.A. & NICOLSON, G.L. (1985). (In preparation).

NORTH, S.M., STYLES, J.M., HOBBS, S.M. & DEAN, C.J. (1982). Monoclonal antibodies to rat sarcomata: I. Immunization procedures and source of lymphoid cells for hybridoma production. *Immunology*, **47**, 397.

READING, C.L., KRAEMER, P.M. & MINER, K.M. (1983). *In vivo* and *in vitro* properties of malignant variants of RAW117 metastatic murine lymphoma/lymphosarcoma. *Clin. Exp. Metastasis*, **1**, 135.

ROBINS, R.A. & BALDWIN, R.W. (1974). Tumour specific antibody neutralization of factors in rat hepatoma-bearer serum which abrogate lymph node cell cytotoxicity. *Int. J. Cancer*, **14**, 589.

SHECVAH, E.M. (1985). The effects of Cyclosporin A on the immune system. *Ann. Rev. Immunol.*, **3**, 397.

SCHIRRMACHER, V., BOSSLET, K., SHANTZ, G., CLOVER, K. & HUBSCH, D. (1979). Tumour metastases and cell mediated immunity in a model system in DBA/2 mice. IV. Antigenic differences between a metastasizing variant and the parental tumour line revealed by cytotoxic T lymphocytes. *Int. J. Cancer*, **23**, 245.

SHIN, H.S., ECONOMOU, J.S., PASTERNAK, G.R., JOHNSON, R.J. & HAYDEN, M.L. (1976). Antibody mediated suppression of grafted lymphoma. *J. Exp. Med.*, **144**, 1274.

STECK, P.A. & NICOLSON, G.L. (1984). Cell surface properties of spontaneously metastasizing rat mammary adenocarcinoma cell clones. *Transplant. Proc.*, **16**, 355.

STUTMAN, O., DIEN, P., WISUN, R.E. & LATTIME, E.C. (1980). Natural cytotoxic cells against solid tumors in mice. Blocking of cytotoxicity by D-mannose. *Proc. Natl Acad. Sci. USA*, **77**(5), 2895.

VAAGE, J. (1973). Humoral and cellular immune factors in the systemic control of artificially induced metastases in C3Hf mice. *Cancer Res.*, **33**, 1957.

WELCH, D.R., MILAS, L., TOMASOVIC, S.P. & NICOLSON, G.L. (1983). Heterogeneous response and clonal drift of sensitivities of metastatic 13762NF mammary adenocarcinoma clones to gamma radiation *in vitro*. *Cancer Res.*, **43**, 6.

WELCH, D.R. & NICOLSON, G.L. (1983). Phenotypic drift and heterogeneity in response of metastatic mammary adenocarcinoma cell clones to Adriamycin, 5-fluro-2′-deoxyuridine and methotrexate treatment *in vitro*. *Clin. Exp. Metastasis*, **1**, 317.

WOODRUFF, M.F.A. & HODSON, B.A. (1985). The effect of passage *in vitro* and *in vivo* on the properties of murine fibrosarcomas. I. Tumourigenicity and immunogenicity. *Br. J. Cancer*, **51**, 161.